# A novel prosthetic foot with a flexible rubber ankle for the Indonesian population: a finite element analysis

**DOI:** 10.1186/s12891-025-09418-w

**Published:** 2025-12-20

**Authors:** Ahmad Khairul Faizin, Ndaru Adyono, Wahyu Dwi Lestari, Abdulfatah Abdu Yusuf, Muhammad Imam Ammarullah

**Affiliations:** 1 Department of Mechanical Engineering, Faculty of Engineering, University of Pembangunan Nasional “Veteran” Jawa Timur, Surabaya, East Java 60294 Indonesia; 2 Department of Mechanical Engineering, College of Engineering, University of Liberia, Monrovia, Montserrado 1000 Liberia; 3 Bioengineering and Environmental Sustainability Research Centre, University of Liberia, Monrovia, Montserrado 1000 Liberia; 4 Department of Mechanical Engineering, Faculty of Engineering, Universitas Diponegoro, Semarang, Central Java 50275 Indonesia

**Keywords:** Finite element analysis (FEA), Prosthetic foot design, Energy return, Flexible rubber ankle, Gait cycle

## Abstract

**Background:**

Prosthetic foot design is crucial for enhancing mobility in individuals with lower limb amputations. However, many existing designs struggle to replicate the natural flexibility and energy return of the human ankle. This study aimed to develop a novel prosthetic foot optimized for the Indonesian population, incorporating a flexible rubber ankle to better mimic human ankle motion and improve energy efficiency.

**Methods:**

The prosthetic foot design featured an aluminum alloy and a rubber filler at the ankle joint. The rubber filler was modeled as a hyperelastic material using the Mooney-Rivlin model. Finite element analysis (FEA) was conducted to simulate quasi-static loading across various stance phases of the gait cycle. Stress distribution and deformation were analyzed to evaluate the prosthetic foot’s performance.

**Results:**

FEA simulations revealed that deformation was primarily concentrated around the ankle, peaking during the terminal stance phase. The rubber ankle demonstrated effective energy absorption and return, with 80.78% of the energy returned during the terminal stance phase. This indicates a high level of efficiency in replicating natural ankle behavior.

**Conclusion:**

The novel prosthetic foot design, featuring a flexible rubber ankle, successfully mimics human ankle flexibility and enhances energy return during gait. This design holds significant potential for improving mobility and comfort for the Indonesian population, particularly during the critical terminal stance phase of walking. Further experimental validation and real-world testing are recommended to confirm its performance and applicability.

## Introduction

According to a study by Barth et al. [1], Indonesia ranked third among 29 countries in the number of persons with amputations (PwA) accessing rehabilitation services provided by the International Committee of the Red Cross (ICRC) from 2009 to 2018. The study also found that the main causes of lower limb amputees in Indonesia were trauma (58.6%), diabetes (24.5%), and congenital defects (9.7%). The average age at amputation for PwA in Indonesia was 32.4 years, lower than the global average of 37.8 years. Unfortunately, there is a lack of research on the prosthetic foot design for the Indonesian population, and most of the available prosthetic feet are imported from other countries or commercial prosthetic foot, which may not meet the local needs and expectations [2, 3].

Recent studies proposed innovative design solutions of prosthetic foot, but the design include artificial ankle was minimally observed [4–7]. According to gait studies, the ankle joint does much more work than any other lower limb joint when walking [8]. Arnell [9] stated that the muscles of the ankle joint generate about 5.4 times more work than they store during walking. In this study, we utilize an artificial ankle incorporating a rubber filler, which has been demonstrated to accommodate large deformations effectively. This design ensures that the structure of the prosthetic foot remains less affected by various loading conditions throughout the stance phases. To further validate the performance and durability of the prosthetic foot, finite element analysis (FEA) was employed. The FEA simulations allowed us to assess stress distribution, deformation patterns, and overall structural integrity under different loading scenarios, ensuring that the prosthetic foot can withstand the complex forces encountered during gait cycles. A successful prosthetic foot design aims to improve load distribution during walking while providing comfort and support in the ankle region. Since the flexibility of the ankle was a crucial parameter during the gait cycle in healthy feet [10]. 

FEA has been applied to various aspects of lower limb prosthetics, such as hip implant [11], socket design [12], and bionic ankle development [13]. Beyond these applications, FEA has been widely utilized in prosthetics for stress-strain analysis [14, 15], evaluation of energy storage and return [15], and verification of compliance with international standards such as ISO 10,328 [16] and American Orthotic and Prosthetic Association (AOPA) Guidelines [17], or equivalent boundary conditions proposed by Tabucol et al. [18]. FEA has also been instrumental in the design and optimization of various prosthetic types, including passive designs [19], semi-active designs [20], and prosthetics optimized for achieving optimal roll-over shapes [21, 22]. Additionally, FEA has been applied to enhance energy-storing-and-returning (ESR) prosthetics with fiberglass [23], compare different prosthetic models [24], and simulate ISO/TS 16,955 standards [25]. The method has also played a critical role in designing 3D-printed prosthetics [26–28], evaluating the effects of temperature on materials [29], and optimizing the shapes and dimensions of foot prosthetics [30]. Notably, Tabucol et al. [18] have demonstrated FEM methodologies to derive key characteristic curves, such as force-displacement and ankle torque-rotation. Furthermore, Leopaldi et al. [31] utilized FEA to optimize a prosthetic foot equipped with a silent block containing rubber at the ankle, enhancing its adaptability to varying terrain levels and conditions. FEA can provide insights into the strains and stresses that occur at the interface between the prosthetic foot and the rubber ankle, which are crucial for the durability and functionality of the prosthesis. FEA can also reduce the reliance on subjective and costly experimental methods [32], and enable the optimization and customization of prosthetic designs for different amputees [33].

Hence, this study used FEA to design and evaluate a novel prosthetic foot for the Indonesian population. The prosthetic foot had an aluminium alloy and a rubber filler at the ankle, which aimed to mimic the human ankle’s flexibility. The rubber filler was a hyperelastic material based on the Mooney-Rivlin model. The FEA simulated the quasi-static loads during the stance phases of gait and analyzed the deformation and stress of the prosthetic foot, focusing on the ankle design performance.

## Methods

This methodology is divided into several sections to provide a more detailed explanation. Section 2.1 describes the geometric construction of the prosthetic foot using 3-dimensional design. Section 2.2 details the evaluation of the required material properties of prosthetic foot components to determine total deformation, stress, strain, and strain energy. Section 2.3 describes the boundary conditions for the numerical study. Section 2.4 describes the finite element model used to evaluate the material characteristics of prosthetic foot.

### Geometry

**Fig. 1:**
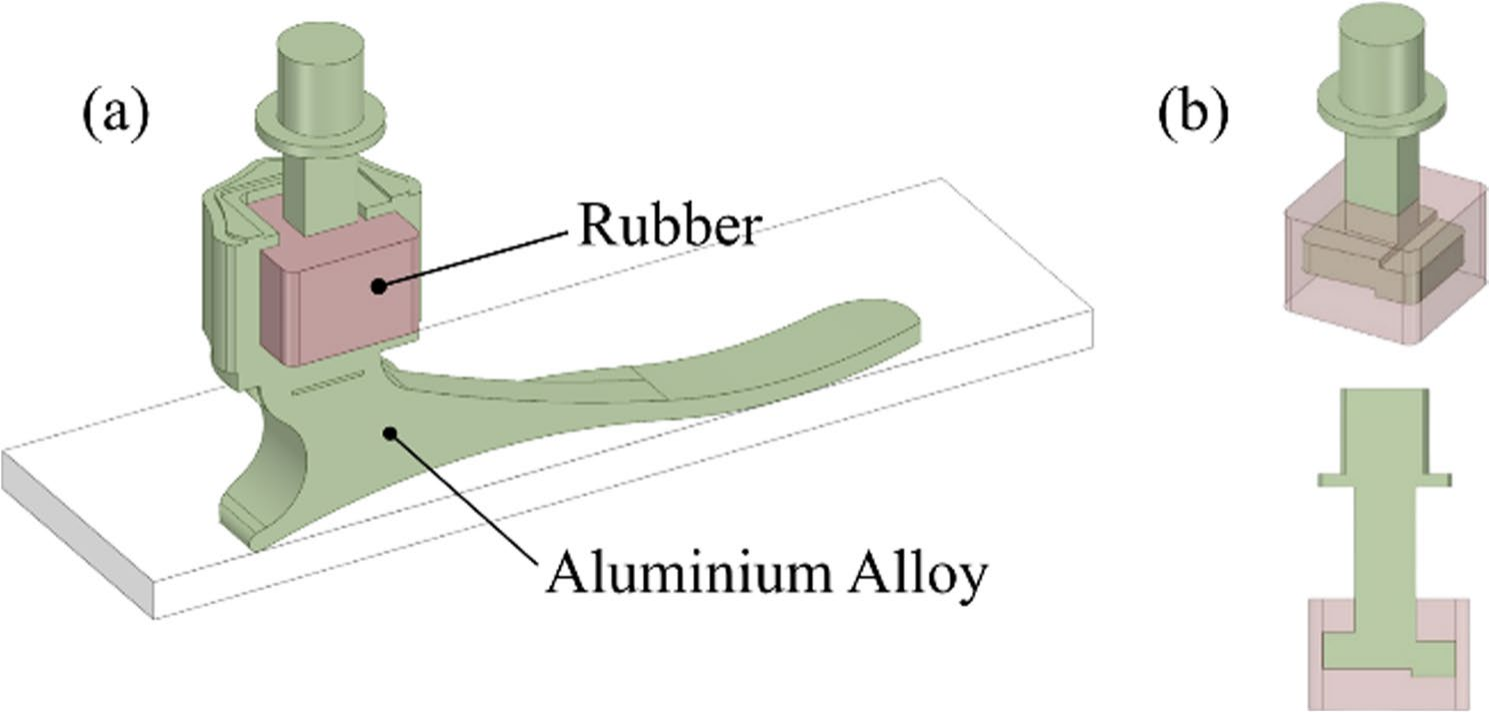
Materials configuration: (**a**) Material of foot prosthesis with rubber filler as artificial ankle and (**b**) Rubber material inclusion in ankle configuration

This study introduces a prosthetic foot, specifically an energy-storing prosthetic foot, based on Indonesian anthropometry. The design of the prosthetic foot in this study was depicted in Fig. 1, which was inspired by Lestari [34] and Chuan et al. [35]. The overall model is presented in Fig. 1(a), while Fig. 1(b) provides a detailed view of the rubber component integrated into the ankle mechanism. This feature aims to imitate a human ankle, helping amputees conserve energy. The rubber inclusion in the ankle configuration is designed to mimic the soft tissues around the human ankle, allowing it to store energy during the stance phase of the gait cycle. This design feature is an original contribution of our study, designed to enhance the functionality and energy efficiency of the prosthetic foot. The energy stored during heel strike is returned to produce a smooth transition from heel strike to foot flat and then heel raise. From the heel rise, the energy stored in the forefoot is then released in a combination of forward (thrust) and upward (lift) directions that complete the gait cycle. This lifting effect helps keep the amputee’s center of gravity at a constant level so that movement is less tiring when conducting daily activities, such as walking.

According to the configuration shown in Fig. 1(b), the force or load extends horizontally from the ankle area posteriorly toward the toe area, while the ankle part features an octagonal shape that extends horizontally and anteriorly from the ankle area. At the ankle, there is also a joint pivot platform used to connect the shank and the ankle. This platform is connected to the octagonal structure using rubber and is square in shape, extending horizontally. At its end, a thin cylinder with a size larger than the square is included to prevent excessive bending at the ankle. This mechanism follows the biomechanical nature of the ankle, as studied by Brockett and Chapman [36].

### Materials

Research in the field of biomedical engineering has explored various materials and techniques for manufacturing foot prostheses, especially ankle ones, including metallic biomaterials [37, 38], additive manufacturing [39–41], orthotics [42], porous structures [43], and light metal alloys [44]. Additionally, research has investigated the influence of ankle torque on the sustainability of joint designs, emphasizing the importance of structural grade aluminum alloys for their high strength to weight ratio [45]. In this study, the foot prosthetic is constructed utilising an aluminium alloy material with density of 2770 kg/m^3^ and tensile yield strength of 280 MPa. Meanwhile, the ankle filler material is rubber with density 1000 kg/m^3^ that imitates ankle movement by pressing the weight during the normal walking phase as shown in Figs. 1(a).

This rubber material mimics the human ankle’s stability and flexibility, making the prosthesis more useful and comfortable. Most research and development in this field focuses on designing and creating prosthetic ankles with soft rubber materials that resemble the human ankle tissues. Since, the soft rubber materials in ankle prostheses helps to replicate ankle properties more accurately, leading to more efficient and comfortable prosthetic devices [13]. This study focuses on providing stability and adaptability for normal walking on flat surfaces, making it particularly suitable for K2 ambulators who require reliable support for everyday mobility. The design ensures effective energy return and flexibility during the gait cycle, addressing the needs of users who walk at slower speeds or for shorter distances.

**Fig. 2:**
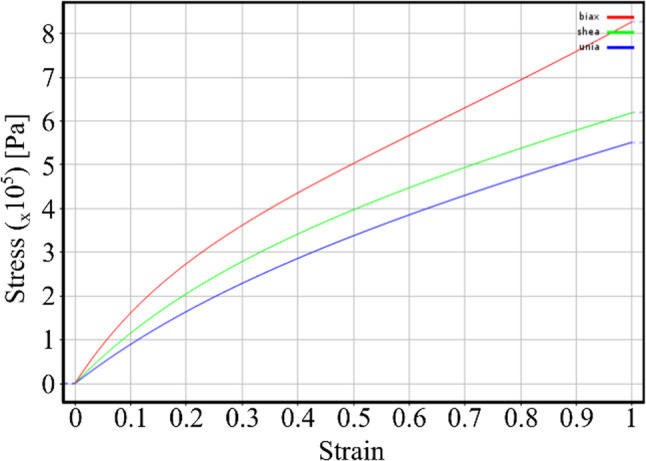
Stress-strain curve of Mooney-Rivlin two-parameter hyperelastic model of rubber

The rubber model in this study using the Mooney-Rivlin two-parameter hyperelastic model provides a crucial understanding of the material’s reaction to deformation. The Mooney-Rivlin (MR) model is a modified version of the neo-Hookean model that includes a Cauchy stress term. The predictions of the Mooney-Rivlin model can be better than those done using the Neo-Hookean model [46]. Equation 1, Eq. 2, and Eq. 3 describe the incompressible MR material, leading to a Cauchy stress in uniaxial deformation, biaxial deformation, and planar deformation. The curve of the Mooney-Rivlin two-parameter model was derived from fitting the experimental data shown in Fig. 2 [47]. The curve is a fundamental representation of the mechanical characteristics of materials. The initial portion of the curve often represents a linear elastic region, where the material experiences elastic deformation and returns to its original shape when the applied force is removed. Once the elastic region is exceeded, the curve transitions into a non-linear phase, demonstrating the material’s hyper-elastic characteristics and ability to withstand higher deformation.

Cauchy stress for Mooney-Rivlin two-parameter under incompressible loading is given by the following equations in 3 common loading modes presented in Eq. 1, Eq. 2, and Eq. 3.

1 $$\:{\sigma\:}_{uniaxial}=2\left({\lambda\:}^{2}-\frac{1}{\lambda\:}\right)\left[{C}_{10}+\frac{{C}_{01}}{\lambda\:}\right]$$

2 $$\:{\sigma\:}_{planar}=2\left({\lambda\:}^{2}-\frac{1}{{\lambda\:}^{2}}\right)\left[{C}_{10}+{C}_{01}\right]$$

3 $$\:{\sigma\:}_{uniaxial}=2{C}_{10}\left({\lambda\:}^{2}-\frac{1}{{\lambda\:}^{4}}\right)+2{C}_{01}\left({\lambda\:}^{4}-\frac{1}{{\lambda\:}^{2}}\right)$$

Where $\:{C}_{01}$ and $\:{C}_{10}$ are material constant; $\:\lambda\:=L/{L}_{0}$ is function of the applied stretch.

### Boundary conditions

**Fig. 3:**
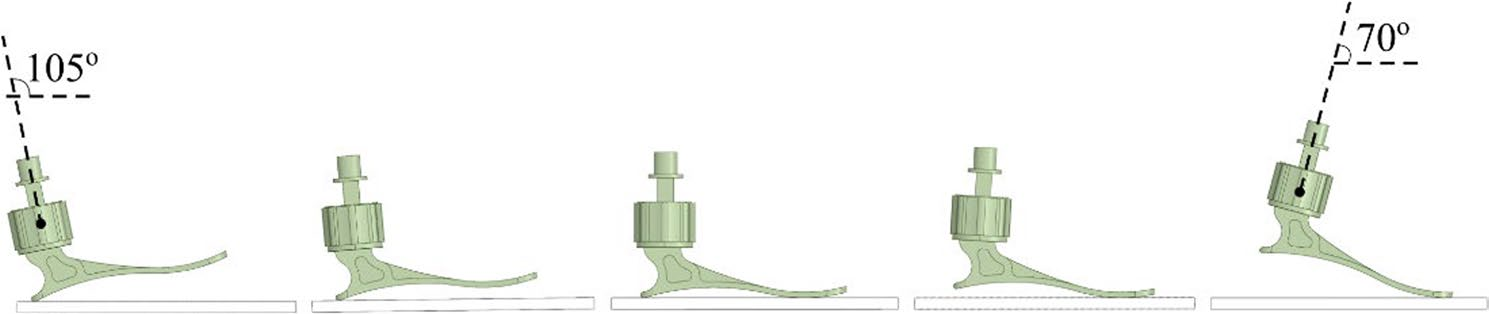
Gait cycle during the stance phases of the prosthetic foot across the angle ranging from 105^o^ to 70^o^ in steps of 10^o^

This study has implemented a quasi-static evaluation in order to evaluate the load on the foot prosthetic in some different positions of stance phases. The top of prosthetic foot underwent an 800 N downward vertical force to represent the weight of body acting from its center of mass. The load is applied increases linearly over time, from 0 N to 800 N. The top of the foot prosthetic was allowed to move vertically while being prevented from moving in the anteroposterior and the mediolateral directions. This restriction was applied because loading was only in the vertical direction, without any horizontal displacement. The bonded fixed support was applied as the boundary condition to constrain the prosthetic foot. This approach was chosen to simplify the simulation and focus on the stress distribution and deformation behavior of the prosthetic foot under quasi-static loading conditions, particularly during the stance phase of gait. These conditions were imposed on positions with top of the foot prosthetic angles ranging from 105° to 70°, in step of 10°, to assess the deformation characteristics of the stance phase of the prosthetic foot, as shown in Fig. 3.

**Fig. 4:**
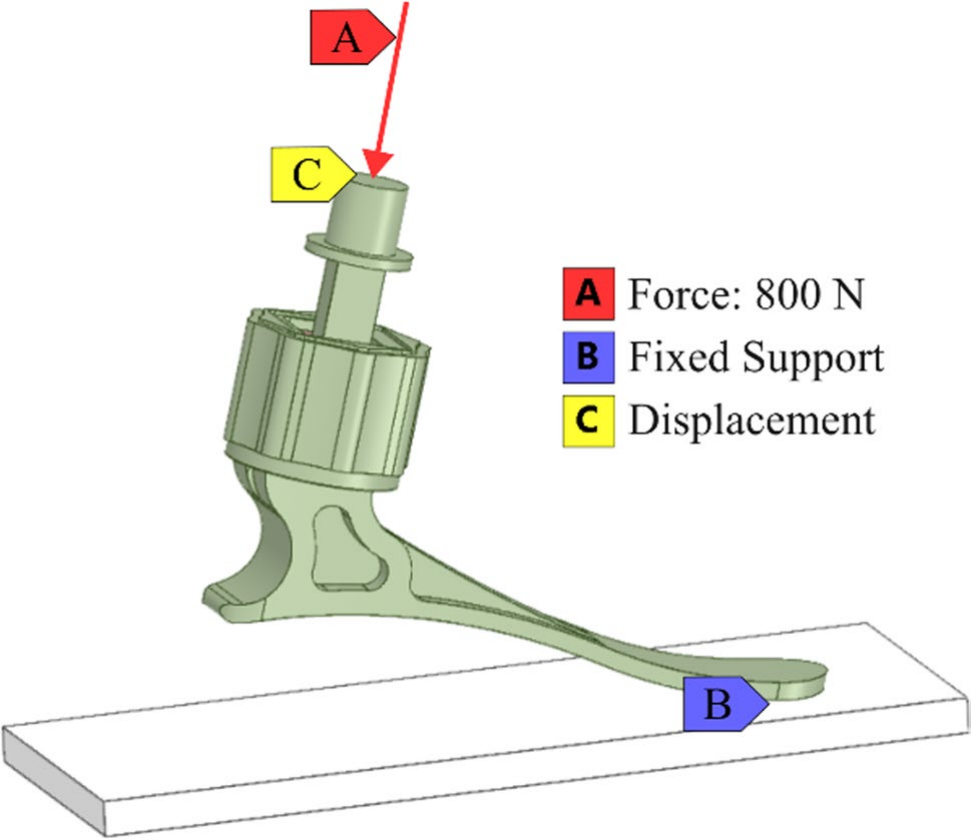
The boundary conditions employed in the model

Figure 4 shows the boundary conditions employed in the built-in finite element model. To accommodate higher-order strains during deformation, the large deflection setup was enabled. The nonlinear equations governing the rubber ankle’s behavior were solved using the line search method of the Newton-Raphson algorithm, with a displacement tolerance of 5%.

### Development of the finite element model

**Fig. 5:**
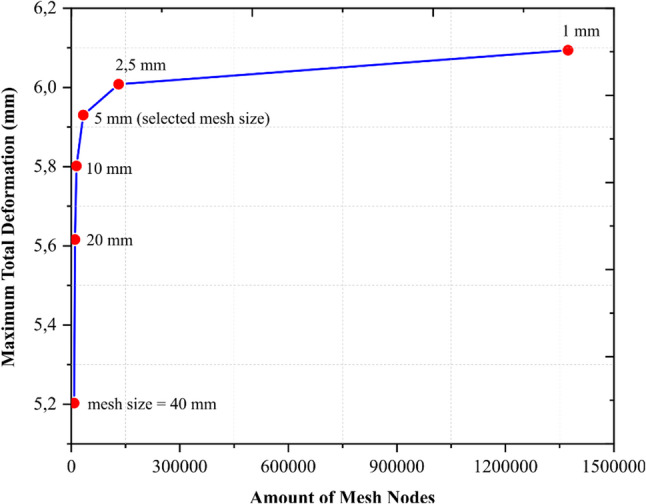
Mesh independent test

Ansys Workbench 19.2 (Ansys Inc., Canonsburg, PA, USA) was used to conduct finite element analysis. The quasi-static analysis was conducted on foot prosthetic geometry for top of foot prosthetic angles ranging from 105° to 70° in step of 10°. A Mooney-Rivlin hyperelastic model was employed to represent the non-linear stress-strain behaviour of rubber filler in ankle part. The Mooney-Rivlin 2 parameters such as material constant *C*_*10*_ of 0.15 MPa, material constant *C*_*01*_ of 0.015 MPa, incompressibility parameter *D*_*1*_ of 0.0012 1/MPa and the rubber density of 1000 kg/m^3^ [47]. The remainder of the components were modelled as isotropic linear elastic material. The foot-ground contact was modelled as frictional, with a coefficient of friction of 0.2 [48]. Bonded contact was the method applied to the remaining contact regions in the setup. A hybrid mesh of 3D 4-noded linear tetrahedron and 8-noded hexahedron elements was used to discretize the domain after the shape of the elements was checked. The mesh independent test was conducted for stance phase at angle of 70° or during the preswing stance, the result was shown in Fig. 5. For all the positions simulated, the mesh nodes and mesh elements had a maximum of 33,236 and 13,859, respectively. This amount of mesh nodes and mesh elements corresponding to the mesh element size 5 mm. The chosen mesh element size of 5 mm was strategically selected to optimize computational efficiency while maintaining acceptable accuracy. This larger mesh size results in a significantly reduced number of nodes, allowing for faster computation times compared to a finer mesh size of 1 mm. In term of response of maximum total deformation, the 5 mm mesh size offers a relative error of only 2.73% to finer mesh size of 1 mm.

## Results and discussion

In this study scenario, selection of the remain material models of the prosthetic foot is aluminum alloys. Since, they have many desirable properties, such as high strength, low weight, good corrosion resistance, and easy fabrication. The prosthesis made of aluminum alloys is considered to have linear properties. Using the rubber ankle filler in this model may help amputees perform activities of daily living by providing additional flexibility, allowing it to more closely mimic the function of a healthy ankle.

**Fig. 6:**
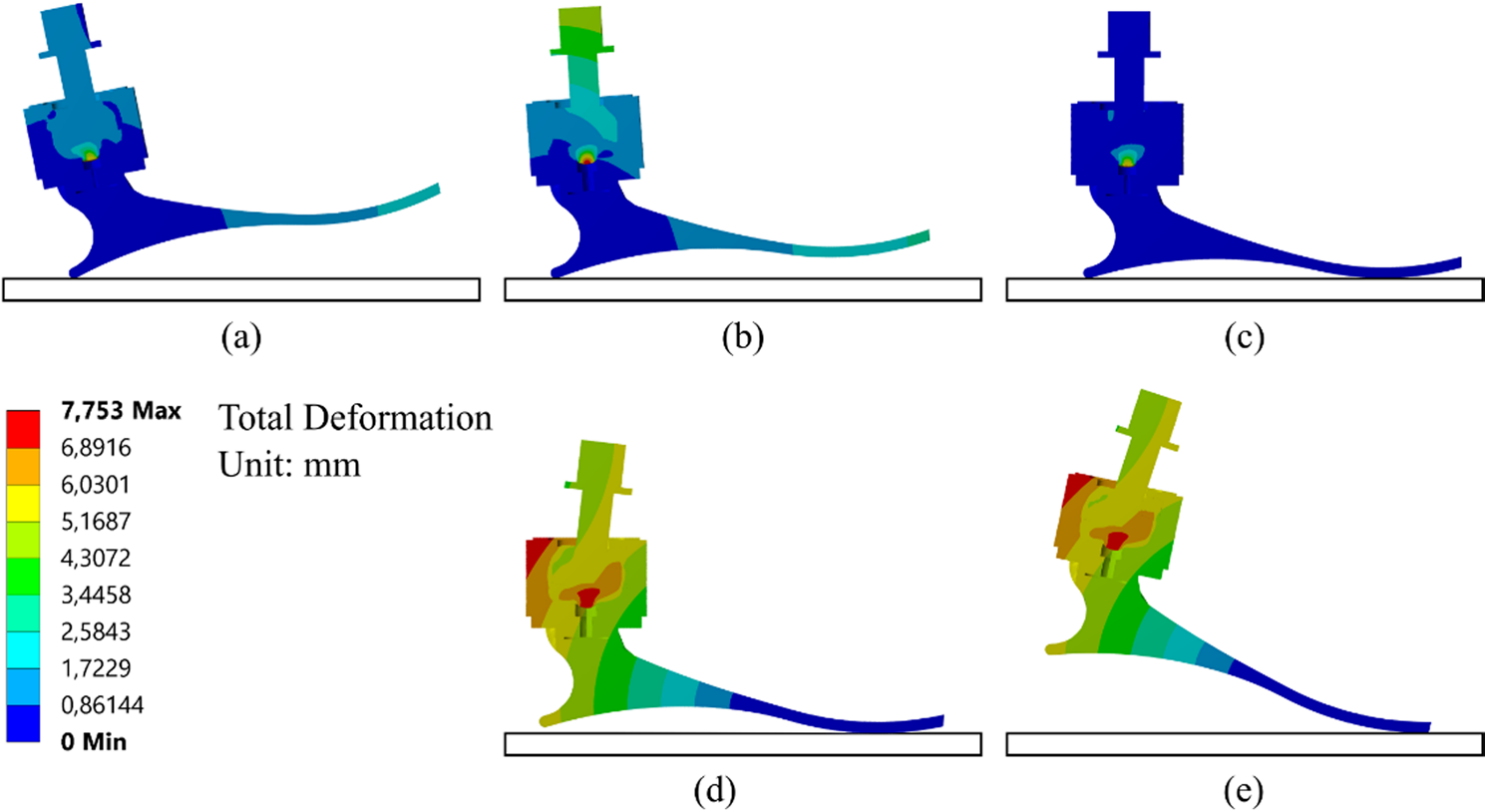
Contour of total deformation across the stance phases of walking: (**a**) Initial contact, (**b**) Loading response, (**c**) Mid-stance, (**d**) Terminal stance, and (**e**) Preswing

**Fig. 7:**
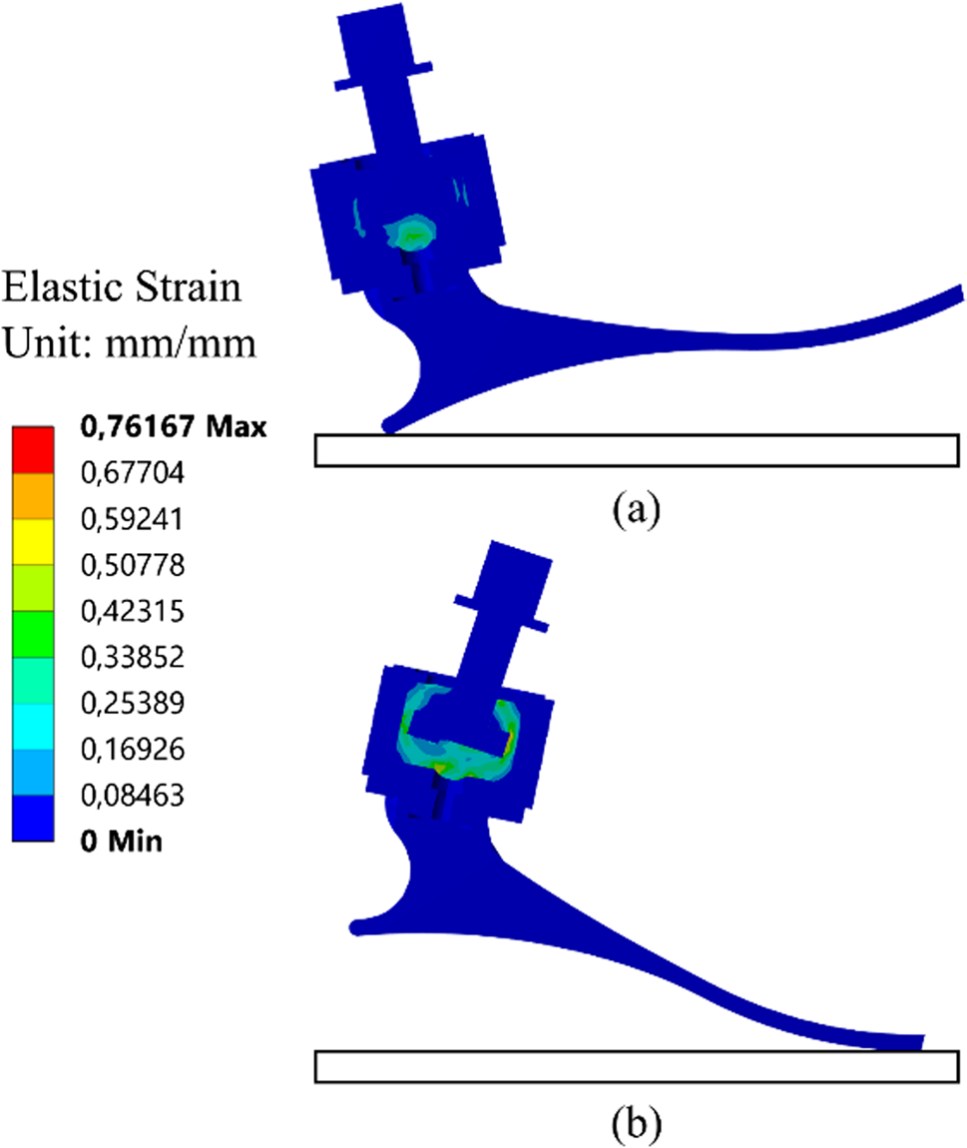
Elastic strain: (**a**) The initial contact and (**b**) The preswing stance

Figure 6 displays the observed deformation characteristics of a prosthetic foot during stance phase of gait cycle. According to numerical results, the prosthetic foot has the maximum deformation during the terminal and preswing stance. Thus, the further analysis was focus on terminal stance. The distribution of von Mises strain during the initial contact stance and the preswing stance is shown in Fig. 7. During the the preswing stance phase, the degree of elongation is greater compared to the initial contact stance due to the presence of an angle that influences the stretching of the rubber filler. Also, the numerical result of strain shown that strain was focus on the rubber rather than in the other part of prosthetic part. This result agrees with our design purpose that the strain should be covered by the rubber filler in ankle. This design agrees with the biomechanics of healthy leg movements, where the joints exhibit more flexibility in the anterior direction [10].

**Fig. 8:**
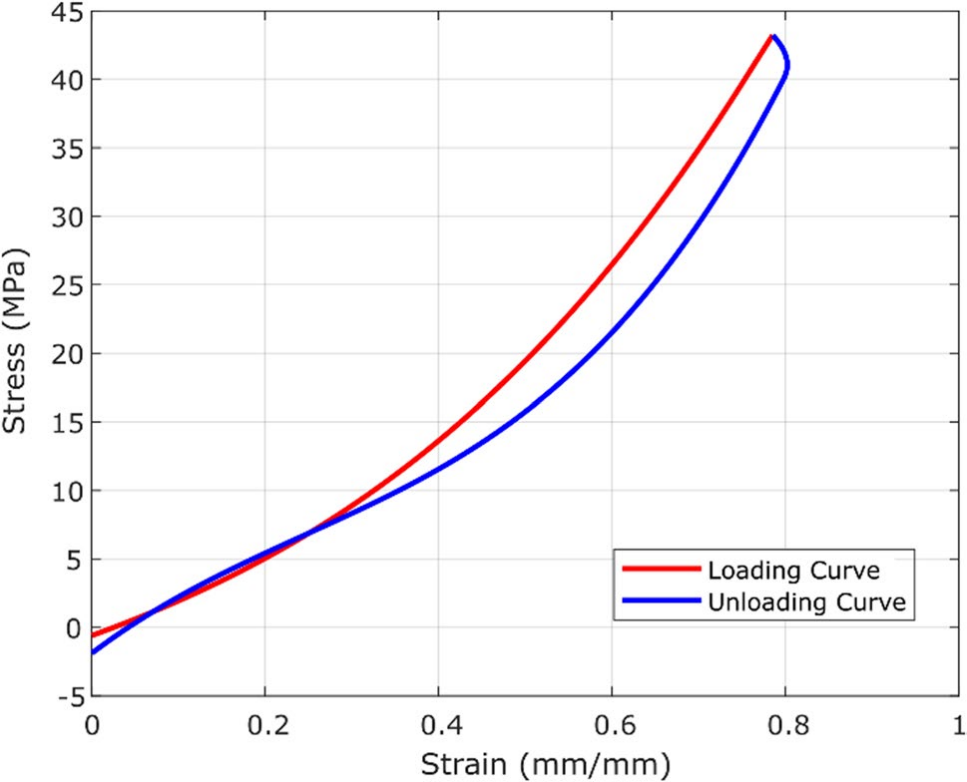
Hysteresis curve of rubber filler as artificial ankle during terminal phase from the numerical result

Figure 8 shows hysteresis curve of rubber filler as artificial ankle during the terminal stance. The stress-strain hysteresis loop in a prosthetic foot, particularly in the context of transtibial prostheses, is an important aspect of understanding the energy storage and return characteristics of the ankle of prosthetic foot [49]. The hysteresis loop represents the energy loss during loading and unloading cycles, and a smaller hysteresis loop indicates a more efficient energy storage and return system in the prosthetic foot. The curve that is positioned below the other curve is considered to have higher resistance [13].

Based on this numerical study, it can be concluded that the prosthesis, which incorporates rubber filler at the ankle, has the capacity to endure stresses of up to 800 N. The hysteresis loop depicted in Fig. 8 exhibits a low magnitude, indicating a good level of efficiency in the prosthesis [50]. The area of the hysteresis loop is 2.47 N mm. The area of the hysteresis loop was calculated using the trapezoid method. The percentage value of the hysteresis loop area according to the area under the load curve is 18.22%, which means 80.78% of the energy was returned by the Money-Rivlin rubber model during the terminal phase and 18.22% of the energy was lost.

The AOPA offers guidelines for fully functional prosthetic foot, including dynamic testing to determine acceptable levels of movement and requirements for proper functioning [51]. Based on the given information, the dynamic testing allows for a displacement of at least 25 mm and a return of at least 75%. For testing purposes that need flexibility, the acceptable amount of displacement is equal to or greater than 25 mm, with a maximum return of 75% [52]. In comparation with this study result, which 80.78% of the energy was returned. The loop area of hysteresis represents the energy loss due to internal friction in the rubber material [53]. While the energy return of 80.78% is promising and exceeds the AOPA’s minimum requirement of 75%, the lower flexibility of the rubber material (indicated by the 18.22% hysteresis loop area) could still pose challenges. For instance, reduced flexibility might lead to discomfort or increased energy expenditure during prolonged use, particularly for active individuals or those walking on uneven terrain. Moreover, the study’s focus on energy return and hysteresis is a critical step, but it should be expanded to include other factors that influence prosthetic foot performance. For example, the durability of the materials under repeated stress, the ability to adapt to different walking speeds, and the impact of varying ground surfaces, such as slopes, stairs, or uneven terrain are all essential considerations. These factors could be incorporated into future dynamic testing protocols to ensure the prosthetic foot performs well in diverse real-life scenarios.

Another area for improvement is the potential trade-off between energy return and flexibility [54]. While the current design prioritizes energy efficiency, it may benefit from a more balanced approach that also enhances flexibility. This could involve experimenting with different rubber compounds or composite materials that offer both high energy return and greater elasticity. For example, materials with a hysteresis loop area closer to 25% might provide a better balance, improving user comfort without significantly compromising energy efficiency.

Additionally, user feedback should be integrated into the evaluation process [55]. Prosthetic foot designs must not only meet technical benchmarks but also address the practical needs and preferences of users. Surveys, gait analysis, and long-term wear tests could provide valuable insights into how the prosthetic foot performs in daily life and whether it reduces the drop-off effect effectively.

This study’s simulation results are encouraging experimental validation is crucial to confirm these findings. Real-world testing under dynamic load conditions, such as walking [56], running [57], climbing stairs [58], or extreme movements [59] would help identify any limitations or areas for improvement. Collaborating with clinicians, prosthetists, and users during this phase could ensure that the design is both functional and user-friendly. The current study demonstrates promising results in terms of energy return, further research is needed to optimize flexibility, durability, and adaptability.

## Conclusions

The primary novelty of this study lies in the development and finite element validation of a prosthetic foot specifically engineered to match the anthropometric data of the Indonesian population. Our work addresses a critical gap by creating a potentially low-cost, high-performance solution tailored to a specific demographic, rather than adapting generic Western designs. The results showed that the design can withstand various loading conditions during stance phases and has a high stiffness-to-weight ratio. The design also incorporates a rubber filler at the ankle part, which can improve the comfort and mobility of the users. This study demonstrates promising results in the development of a prosthetic foot, particularly in terms of energy return, which exceeds the AOPA’s minimum requirement of 75% with an achieved return of 80.78%. However, the lower flexibility of the rubber material, indicated by a hysteresis loop area of 18.22%, suggests room for improvement to enhance user comfort and adaptability. Instead of this study lays a strong foundation for the development of high-performance prosthetic feet, the simulation results are encouraging, experimental validation under real-world conditions and dynamic load scenarios is essential to confirm the prosthetic foot’s performance and durability. Future work should focus on optimizing the material properties to balance energy return and flexibility, exploring alternative materials with lower hysteresis loop areas, and testing the design under diverse conditions such as uneven terrain, varying walking speeds, and prolonged use. Additionally, incorporating user feedback and clinical evaluations will ensure that the prosthetic foot not only meets technical benchmarks but also addresses the practical needs of users.

## Data Availability

All data generated or analyzed during this study are included in this published article. No additional datasets were generated or analyzed beyond the contents of the manuscript.

## Abbreviations

AOPA: American Orthotic and Prosthetic Association (AOPA)
ESR: Energy-storing-and-returning
FEA: Finite element analysis
ICRC: International Committee of the Red Cross
PwA: Persons with amputations
